# An X-ray diffraction study on a single rod outer segment from frog retina

**DOI:** 10.1107/S0909049512018535

**Published:** 2012-05-18

**Authors:** Naoto Yagi, Tatsuhito Matsuo, Noboru Ohta

**Affiliations:** a Japan Synchrotron Radiation Research Institute, SPring-8, 1-1-1 Kouto, Sayo-cho, Sayo-gun, Hyogo 679-5198, Japan

**Keywords:** retina, microbeam, lamellar diffraction

## Abstract

X-ray diffraction patterns were recorded from isolated single rod outer segments of frog. The outer segments in Ringer’s solution were exposed to a 6 µm microbeam (15 keV) at the BL40XU beamline, SPring-8. The diffraction pattern demonstrated a remarkable regularity in the stacking and flatness of the disk membranes. The electron density profile calculated from the intensity of up to tenth-order reflections showed a pair of bilayers that comprise a disk membrane. The structure of the disk membrane and the changes in the profile on swelling generally agreed with previous reports. Radiation damage was significant with an irradiation of 5 × 10^5^ Gy which is much lower than the known damaging dose on proteins at the liquid-nitrogen temperature.

## Abstract 2

X-ray diffraction was recorded from retinal rod outer segments of frog using a microbeam.

## 1. Introduction   

**Figure 1:**
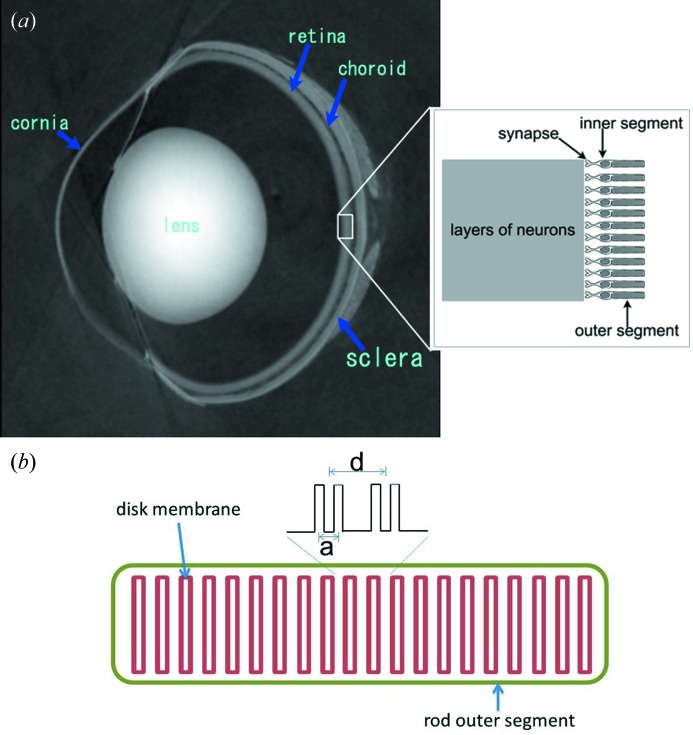
(*a*) Image of the central section through a frog eye obtained by phase-contrast X-ray tomography using a Talbot grating interferometer (Hoshino *et al.*, 2011 ▶). The rod is located at the outer rim of the retina and the outer segment is pointing away from the light. (*b*) Schematic drawing of the arrangement of the disk membranes in a rod outer segment. Each disk membrane comprises of a pair of bilayers. *d* is the inter-disk spacing, while *a* is the intra-disk distance, *i.e.* the distance between two bilayers in one disk. Since *d* is about 30 nm and the length of the rod outer segment is about 50 µm, the number of disk membranes is about 1500. The electron density profile across two disk membranes is represented schematically.

The vertebrate retina is an imaging device that lies on the inner surface of the eye (Fig. 1*a* ▶). The retina has a layered structure and the photosensors are rods and cones that are in the back of retina, facing the choroid. Rods are generally larger than cones and more abundant in frog retina. The rod outer segment (ROS) is the distal part of the rod. It has a cylindrical shape, about 6 µm in diameter and about 50 µm long, and about 1500–2000 disk membranes are regularly stacked in the cytoplasm along the length of the outer segment (Fig. 1*b* ▶). The disk membrane is produced by envacuolation of the plasma membrane and thus it is a flattened closed vesicle. Each disk membrane comprises of a pair of lipid bilayers (made mostly of phospholipids and cholesterol) that was formerly a part of the plasma membrane. Each bilayer contains a large amount of photoreceptor protein rhodopsin, occupying about half of the surface area, that spans the entire bilayer of the disk membrane. The chain of reactions in the ROS to light illumination is briefly illustrated as follows. Light is absorbed by a 1-*cis*-retinal in rhodopsin, causing a conformational change in rhodopsin that activates G-protein transducin. Transducin activates cGMP (cyclic guanosine monophosphate-specific phosphodiesterase), leading to reduction of the cytoplasmic cGMP concentration. Since the cation channel in the plasma membrane of the ROS is activated by cGMP, the reduction in the cytoplasmic cGMP concentration leads to closure of the channel, hyperpolarizing the membrane. This creates a signal that is conducted to the synapse which is located in the inner segment. The signal is transmitted through the neural network in the retina and optic nerve to the visual cortex in the brain.

X-ray diffraction studies on the ROS were carried out extensively about 40 years ago (Blaurock & Wilkins, 1969 ▶; Chabre & Cavaggioni, 1973 ▶; Corless, 1972 ▶; Gras & Worthington, 1969 ▶). Strong oriented X-ray diffraction was observed from the ROSs and the electron density profile across the disk membrane was obtained. In a retina or isolated ROSs, light-induced structural change in the disk membrane was studied (Chabre, 1975 ▶; Chabre & Cavaggioni, 1973 ▶; Corless, 1972 ▶). Neutron diffraction studies were also conducted on the light-induced structural change (Saibil *et al.*, 1976 ▶; Yeager *et al.*, 1980 ▶).

Although molecular and cellular mechanisms of photo-transduction have been considerably better understood over the last three decades, as far as the authors are aware an X-ray diffraction study on the ROS has not been reported after these early experiments. Thus, intense X-rays from synchrotron radiation, which became available in the late-1970s, have not been used to study the structure of the ROS. In particular, an X-ray beam with a diameter of 6 µm became available recently at the SPring-8 synchrotron radiation facility (Ohta *et al.*, 2005 ▶). Since this beam size is similar to the diameter of the ROS, recording diffraction from an isolated single ROS was attempted. The resultant diffraction pattern shows the remarkable regularity of the disk membranes.

## 2. Materials and methods   

### 2.1. Specimen   

Rod outer segments were obtained from a bullfrog (*Rana catesbeiana*) eye. The retina was removed from the eye and gently shaken in a small volume of frog Ringer’s solution (115 m*M* NaCl, 2.5 m*M* KCl, 1.8 m*M* CaCl_2_, 3.0 m*M* Hepes, pH adjusted to 7.2 at 298 K). Rods were readily broken at the cilia and ROSs floated in the Ringer’s solution together with vitreous humour. For X-ray diffraction studies a drop of the suspension was sandwiched between two sheets of Mylar (6 µm thick) and sealed to avoid evaporation. The gap between the two Mylar sheets was 0.3–0.5 mm, so that the suspension could move freely. In the X-ray measurements the sample was set vertically on a motorized stage and the X-ray beam passed through the two Mylar sheets. The ROSs that attached weakly to the Mylar sheets were examined. Samples were handled under the ambient light. The ROSs were studied for up to 2 h after separation from the retina. Experiments were conducted in accordance with the regulations of the SPring-8 Animal Care and Use Committee.

### 2.2. X-ray techniques   

Microbeam X-ray diffraction experiments were made at beamline BL40XU at the SPring-8 synchrotron radiation facility (Hyogo, Japan) (Inoue *et al.*, 2001 ▶). The peak X-ray energy was 15.0 keV. The energy bandwidth was about 3%. The X-ray beam was passed through a 5 µm pinhole to create a microbeam (Ohta *et al.*, 2005 ▶). The beam size at the specimen, which was 70 mm from the pinhole, was about 6 µm in diameter. The sample-to-detector distance was about 1500 mm. The Bragg spacing was calibrated with powder diffraction from silver behenate (orders of the 001 reflection at 1/5.838 nm^−1^). The X-ray detector was an image intensifier with a beryllium window (V5445P, Hamamatsu Photonics, Hamamatsu, Japan) coupled to a cooled CCD camera (ORCA-II-ER, Hamamatsu Photonics). The pixel size was ∼0.13 mm × 0.13 mm, the X-ray flux was ∼5 × 10^11^ counts s^−1^ and the beam size at the detector was ∼0.1 mm. A fast X-ray shutter that worked in milliseconds was used to avoid unnecessary radiation on the sample. On the X-ray camera the sample was observed with a microscope and a ROS was located. Then the sample was moved either horizontally or vertically in 5 µm steps so that the ROS moved across the X-ray beam. X-ray diffraction patterns were recorded at each position. The experiment was performed at the room temperature (300 K).

### 2.3. Data analysis   

**Figure 2:**
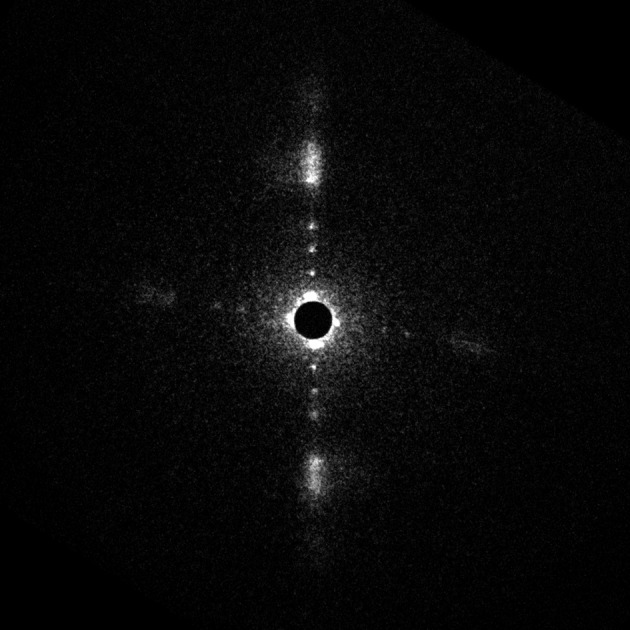
X-ray diffraction patterns from isolated ROSs of frog. The beam is passing through a ROS that lies vertically. The beam also passed through a part of another ROS that is horizontally oriented. The background is not subtracted in this image. The exposure time was 50 ms. The disk spacing of the vertical lamellar pattern is 30.4 nm.

To remove the background a diffraction pattern obtained just outside of a ROS was subtracted from that obtained from the ROS. The intensity in the direction perpendicular to the plane of the disk membranes (meridian) was integrated to obtain a one-dimensional intensity distribution. In a well oriented diffraction pattern like that in Fig. 2, this is equivalent to integration across the meridian. The intensity profile was fitted with a sum of ten Gaussian peaks representing ten orders of reflections. The positions of the ten peaks were determined by a single parameter, the disk spacing [*d* in Fig. 1(*b*) ▶]. The widths of the peaks were determined by a single parameter with the assumption that the width increases with the square of the order of the reflection (Schwartz *et al.*, 1975 ▶). This assumption seems valid because it gave a much better fit to the data compared with an assumption that the width increases linearly with the order. The size of the X-ray beam at the detector (about 100 µm) and the 3% bandwidth of the X-ray beam were taken into account. Other parameters were the peak heights of the first to the tenth reflections. The curve fitting was performed by a least-squares method using the modified Levenberg–Marqualdt algorithm. The initial parameter for the height was the peak height in the observed intensity profile, and the fitting program generally converged without changing the peak heights for the first to the fifth reflections. On the other hand, there are considerable overlaps between neighboring peaks in the sixth to the tenth reflections, and thus the estimated height was generally lower than the peak height.

For the calculation of the electron density profile of the disk membrane, the integrated intensity of each reflection was multiplied by a Lorenz factor that is proportional to the order of the reflection, and its square root was used for the amplitude. Since the disk membrane comprises a pair of centrosymmetrically arranged bilayers, the phases of the reflections are either 0 or 180°. The phase combination obtained from swelling experiments, which was used in previous studies (Chabre & Cavaggioni, 1973 ▶; Corless, 1972 ▶), *i.e.* +−+++−−−++, was used. It should be pointed out that, from a neutron diffraction study, Yeager *et al.* (1980 ▶) concluded that the sign of the first order should be a minus sign for the X-ray data.

## 3. Results and discussion   

Fig. 2 ▶ shows an X-ray diffraction pattern recorded from a ROS with a 6 µm beam. Four sharp diffraction spots and broader peaks were observed in the direction of the long axis of the ROS (meridian). The beam was passing an edge of a second ROS that gives rise to a weaker but similar pattern in the other direction. There are about 200 disk membranes in the 6 µm beam. Compared with the previously obtained diffraction patterns recorded with a point focus beam (Blaurock & Wilkins, 1969 ▶; Chabre, 1975 ▶), it is clear that the angular broadening of the peaks observed in the previous studies was mostly due to misalignment of the ROSs in the sample. On the other hand, the broadness of the sixth and seventh orders along the meridian is still distinct in this pattern, showing that this broadening is not due to variation of the lamellar spacing among ROSs but to that within each ROS.

The diffraction pattern in Fig. 2 ▶ was obtained with a 50 ms exposure. With a longer exposure the sharp diffraction spots were not observed, showing that radiation damage takes place. With an exposure time shorter than 50 ms there was not a significant difference in the relative intensity of the reflections. The dose in a 50 ms exposure was about 5 × 10^5^ Gy. This is lower than the so-called Henderson limit of 2 × 10^7^ Gy (Henderson, 1990 ▶). Damage is also observed with a similar or shorter exposure in intact frog skeletal muscle (Yagi, 2003 ▶). Since the Henderson limit is for cryogenically cooled protein crystals, the radiation damage must be very severe at room temperature. In the present experiment this radiation damage hampered investigation on the diffraction at higher angles or the in-plane diffraction that appears in the equatorial direction. In fact, the in-plane diffraction that has been reported (Blasie *et al.*, 1969 ▶; Blaurock & Wilkins, 1972 ▶) was undetectable in the present experiment.

**Figure 3:**
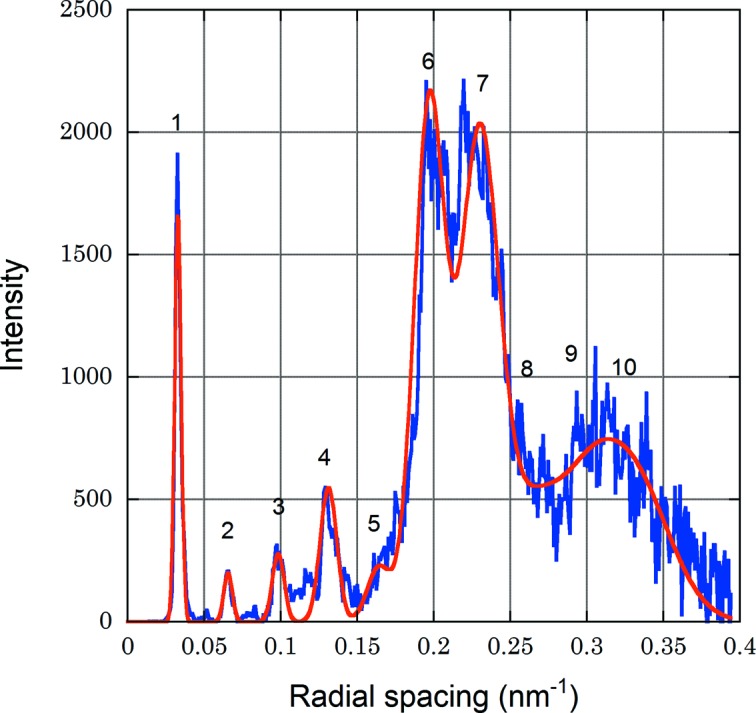
Intensity distribution in the direction along the length of the rod outer segment obtained from Fig. 2 ▶. The black curve (red online) is the background-subtracted experimental data after the Lorentz correction and the grey curve (blue online) is the fitted intensity. The intensity is on an arbitrary scale. The disk spacing is 30.4 nm.

**Figure 4:**
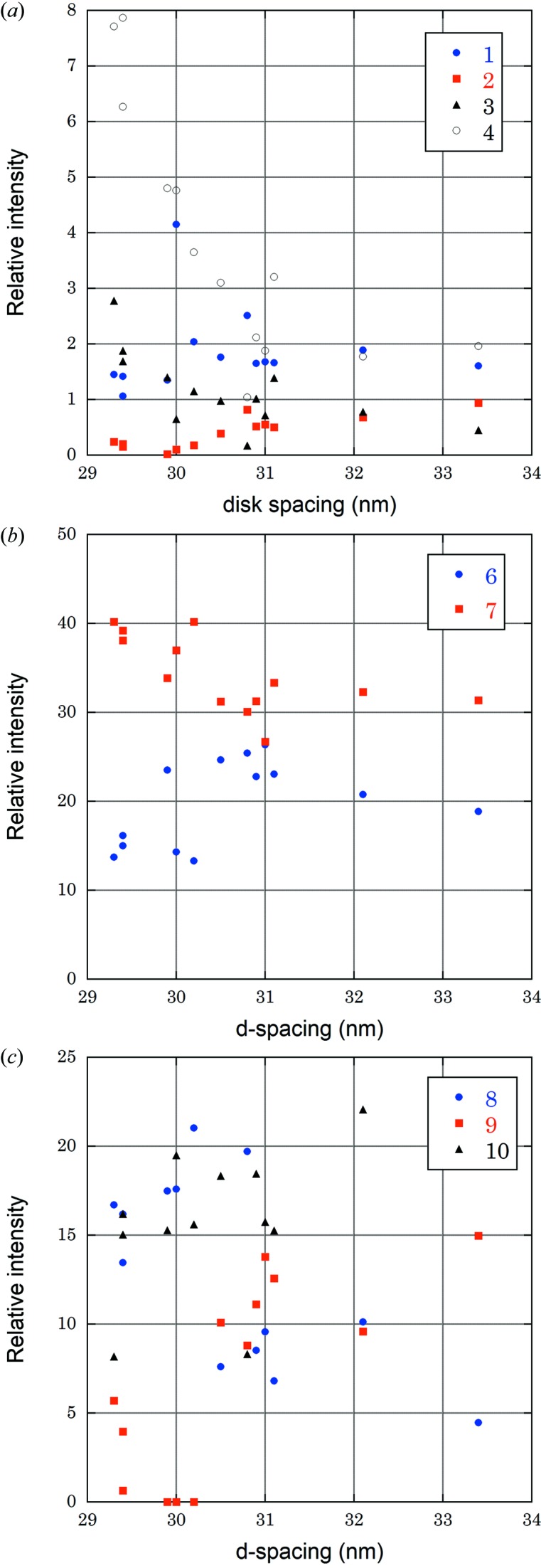
Dependence of the intensity of the lamellar reflections on the disk spacing. (*a*) The first, second-, third- and fourth-order reflections. (*b*) The sixth- and seventh-order reflections. (*c*) The eighth-, ninth- and tenth-order reflections. The fifth reflection was too weak to quantify. The integrated intensity of each order after correction with the Lorentz factor is given as a percentage of the sum of intensities of all ten reflections. The data from 13 ROSs are plotted.

The intensity profile along the meridian was generally similar to that obtained previously from a retina or an isolated ROS (Chabre, 1975 ▶; Chabre & Cavaggioni, 1973 ▶). The ten Gaussian peaks fitted the experimental profile reasonably well (Fig. 3 ▶). The full width at half-maximum of the first-order reflection was only 0.7 pixels, which is approximately the beam size at the detector. This indicates that the periodicity persists for at least a few micrometers, across the entire beam. It also demonstrates that the X-ray is coherent within the beam. Among the individual 13 ROSs analyzed in this experiment, the *d*-spacing (center-to-center distance between the neighboring disk membranes) varied between 29 and 34 nm. This variation is not due to the method of preparation of the ROSs because it was observed in the same batch of ROSs from the same retina. Although not fully investigated, swelling seemed to proceed with time in some ROSs (Bownds & Brodie, 1975 ▶). This may be due to accumulation of Na^+^ within the cell after the inner segment with Na-K ATPase was removed. Under ambient light there is still an influx of Na^+^ but it is not removed from the ROS when the inner segment is lost. The minimum disk spacing observed was 29.4 nm which is close to the reported value for the ROS in intact eye (29.5 ± 0.5 nm; Webb, 1972 ▶). When the disk spacing increased, the intensity of some orders changed in a systematic manner: the fourth, seventh and eighth orders decreased while the second, sixth and ninth increased (Fig. 4 ▶).

The peaks from the disk membranes are also very small in the direction perpendicular to the meridian (equatorial direction). For the first four orders the spot size was almost the same as the beam size. The sixth and seventh orders show some broadening in the equatorial direction, but they are in fact less broad in the equatorial direction than in the meridional direction. These observations show that the disk membranes are remarkably flat and stacked well. Although a periodic lamellar structure has been observed by electron microscopy, the level of regularity demonstrated here exceeds that observed with a modern electron microscopy technique (Nickell *et al.*, 2007 ▶).

**Figure 5:**
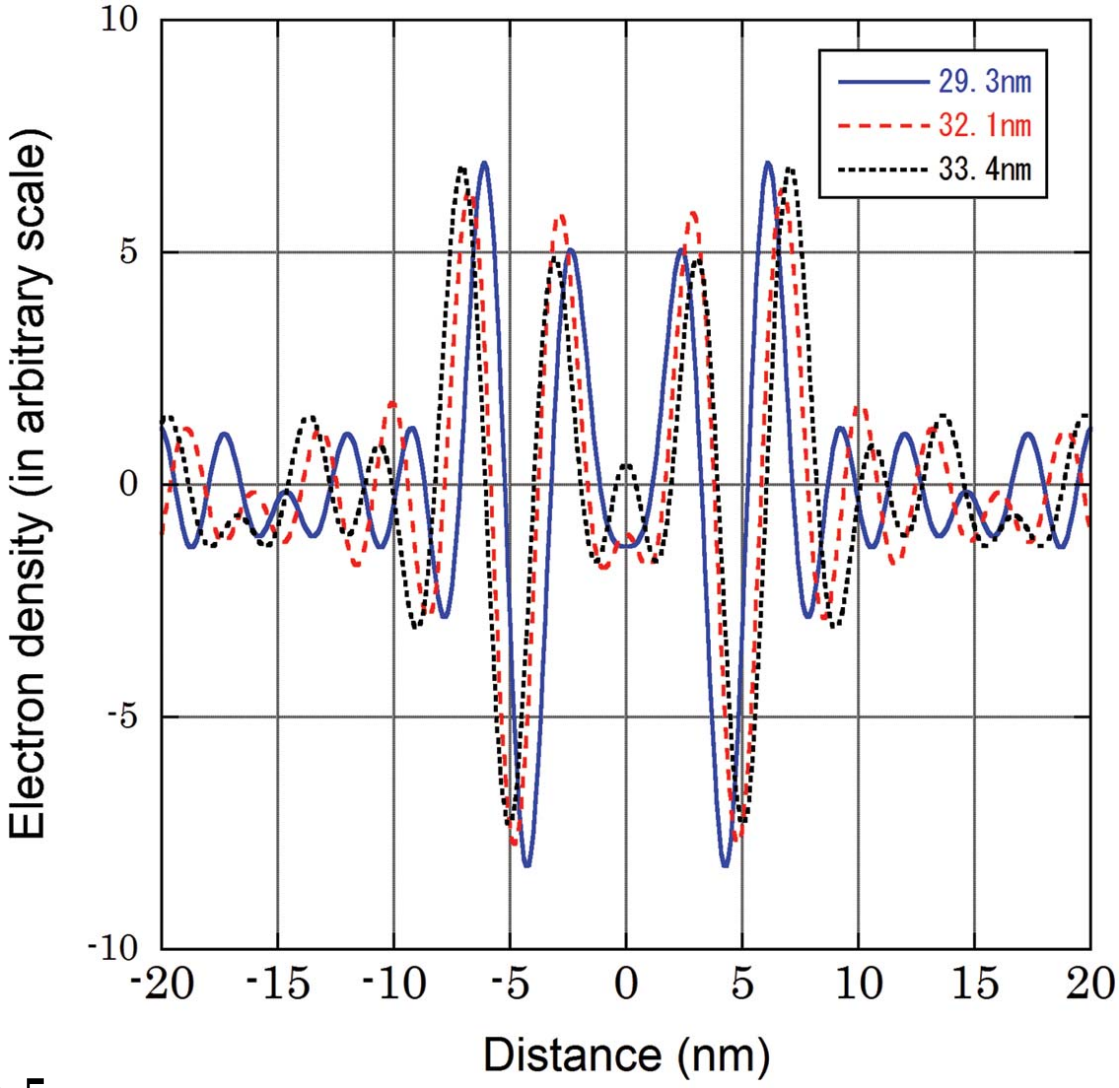
Electron density profile calculated from the experimental data in Table 1 ▶. Profiles from the data with different disk membrane spacing are shown. For the profile at 29.3 nm, data from three ROSs were averaged. The center is that of the disk membrane. The fine ripples are due to truncation of the reflections used to calculate the density at the tenth order.

The data from the three ROSs with a lamellar spacing of 29.3 nm were averaged (Table 1 ▶) to calculate the electron density profile of the disk membrane (Fig. 5 ▶, blue online ‘29.3 nm’ solid curve). It shows symmetrically arranged two bilayers, each of which has a low-density region flanked by two high-density regions. These two bilayers form a disk membrane. The low-density region at the center of the bilayer corresponds to the hydrocarbon chains while the two peaks are due to the phosphate groups of phospholipids. The ripples in the profile are mostly artifacts due to truncation of the reflections at the tenth order, although there is a possibility that part of the ripples represents membrane-associated proteins. The distance between the two peaks in the bilayer, which corresponds to the separation between phosphate groups in phospholipids, was 3.7 nm. This is smaller than the distance measured in synthetic phospholipid–cholesterol bilayers (4–5 nm; Chen *et al.*, 2007 ▶). The difference may be due to the presence of a large amount of rhodopsin in the disk membrane. In the profile in Fig. 5 ▶ the outer peak of the bilayer is higher than the inner peak. It is considered that the proteins that play roles in the signal transduction, such as transducin and phosphodiesterase, are weakly associated with the disk membrane. Thus, these proteins may contribute to this density, but, as most previous studies showed a symmetric profile (Blaurock & Wilkins, 1969 ▶; Chabre, 1975 ▶; Corless, 1972 ▶), we have to be cautious in the interpretation.

Compared with previous studies which also used up to the tenth orders (Blaurock & Wilkins, 1969 ▶; Chabre, 1975 ▶; Corless, 1972 ▶), the profile in Fig. 5 ▶ has larger ripples arising from truncation of reflection orders. The intensity obtained in the present study is generally low in the low orders (especially the first) and high in the higher orders (eighth to tenth). For quantitative comparison, the only intensity values available in the literature are those by Corless (1972 ▶). Assuming the values given by Corless were before Lorentz correction, the intensity of the first reflection after the correction was larger than 10% of the sum of the ten orders, while it is only 1–2% in the present study (Table 1 ▶). On the other hand, the eighth to tenth orders were only less than 10% of the sum, while the eighth and tenth are more than 10% in the present study. A possible cause of the difference is the use of a line-focus X-ray beam in the previous studies. As for the first-order reflection, it may be too sharp to measure its intensity correctly. Ideally, the diffraction pattern should be recorded while the ROS is continuously tilted towards the X-ray beam across the right angle, but this is difficult in the present experiment. The variation of the first-order intensity in Fig. 4(*a*) ▶ does not suggest that the reflection can be completely off the Ewald sphere. It should be noted that the bandwidth of the X-rays used in this experiment was about 3% so that the Ewald sphere was thicker than in other studies.

When swelling of 14% took place from a *d*-spacing of 29.3 to 33.4 nm, the distance between the central bottoms of the two bilayer membranes within a disk membrane, which was 8.6 nm at *d* = 29.3 nm, also increased by 14% (Fig. 5 ▶). This shows that the interior of the disk is not connected to the outside of the cell, and there is a mechanism to adjust to the osmotic balance across the bilayers of the disk. On the other hand, the peak-to-peak distance within the bilayer membrane [*a* in Fig. 1(*b*) ▶, 3.7 nm at *d* = 29.3 nm], which corresponds to the distance between phosphate groups, increased by only 8%. Thus, the swelling took place with a smaller effect on the structure of the bilayer membrane. At a disk spacing of 32.1 nm, intermediate changes were observed. These observations are consistent with the previous conclusions from the osmotic experiment (Blaurock & Wilkins, 1972 ▶).

In summary, the present experiment with a micro X-ray beam showed that (i) the disk membranes in a ROS are ordered with a remarkable regularity and the characteristics of the intensity profile, especially the broadening above the sixth order, are intrinsic in each ROS, (ii) swelling may take place after the ROSs are removed from intact rods, and (iii) radiation damage takes place with an X-ray exposure of the order of 10^5^ Gy. The observed electron density profile and the changes in the disk membrane structure due to swelling are compatible with previous reports. Although the radiation damage limits more detailed structural studies, the present results demonstrate the power of the X-ray microbeam from synchrotron radiation. With an X-ray free-electron laser there is a possibility that diffraction can be recorded before radiation damage takes place (Neutze *et al.*, 2000 ▶). Thus, it may be possible to acquire data at higher spatial resolution and also to investigate responses of a single photoreceptor cell to light.

## Figures and Tables

**Table 1:** Integrated intensities of the orders of reflections that were used to calculate the electron density profiles in Fig. 5 ▶; these were taken from Fig.4 ▶ Values are normalized so that the sum of all orders is 100. The values at *d* = 29.3nm are averages of three ROSs.

	Order									
	1	2	3	4	5	6	7	8	9	10
*d* = 29.3nm	1	0	2	7	3	15	39	15	3	13
*d* = 32.1nm	2	1	1	2	0	21	32	10	10	22
*d* = 33.4nm	2	1	0	2	4	19	31	4	15	21
